# Are Individual Differences in Arithmetic Fact Retrieval in Children Related to Inhibition?

**DOI:** 10.3389/fpsyg.2016.00825

**Published:** 2016-06-14

**Authors:** Elien Bellon, Wim Fias, Bert De Smedt

**Affiliations:** ^1^Psychology and Educational Sciences, Parenting and Special Education Research Group, Katholieke Universiteit LeuvenLeuven, Belgium; ^2^Experimental Psychology, Ghent UniversityGhent, Belgium

**Keywords:** mathematical competencies, individual differences, arithmetic fact retrieval, inhibition, numerical magnitude processing, third grade

## Abstract

Although it has been proposed that inhibition is related to individual differences in mathematical achievement, it is not clear how it is related to specific aspects of mathematical skills, such as arithmetic fact retrieval. The present study therefore investigated the association between inhibition and arithmetic fact retrieval and further examined the unique role of inhibition in individual differences in arithmetic fact retrieval, in addition to numerical magnitude processing. We administered measures of cognitive inhibition (i.e., numerical and non-numerical stroop tasks) and a complementary, more ecologically valid measure of children’s inhibition in the classroom (i.e., teacher questionnaire), as well as numerical magnitude processing (i.e., symbolic and non-symbolic numerical magnitude comparison) and arithmetic fact retrieval (i.e., two verification tasks) in 86 typically developing third graders. We used a correlation, a regression and a Bayesian analysis. This study failed to observe a significant association between inhibition and arithmetic fact retrieval. Consequently, our results did not reveal a unique contribution of inhibition to arithmetic fact retrieval in addition to numerical magnitude processing. On the other hand, symbolic numerical magnitude processing turned out to be a very powerful predictor of arithmetic fact retrieval, as indicated by both frequentist and Bayesian approaches.

## Introduction

There are large individual differences in the way children acquire mathematical competencies (e.g., Dowker, 2005). Because mathematical skills are crucial abilities in modern western society (e.g., Finnie and Meng, 2001; Ancker and Kaufman, 2007) and early individual differences in mathematics predict later adult socioeconomic status (Ritchie and Bates, 2013), it is important to understand the cognitive processes underlying children’s achievement in mathematics as this can contribute to designing scientifically validated diagnostic tests and remediation programs for children at risk for or with difficulties in mathematical achievement. One way to do so, is to investigate the cognitive determinants of these individual differences.

Since mathematics consists of various different abilities (e.g., arithmetic, problem solving, geometry; e.g., Gilmore et al., 2013) it is important to determine how cognitive determinants are related to specific mathematical skills when studying individual differences in mathematical abilities. Most existing studies focussed on associations with general tests of mathematical abilities (De Smedt et al., 2013), which yield a total score that reflects performance averaged across various math abilities. This leaves it functionally unclear how cognitive determinants are involved in mathematical processes. In this study, we focus on arithmetic, and more specifically arithmetic fact retrieval. The ability to retrieve arithmetic facts is a major building block for children’s development in more complex mathematical abilities (Kilpatrick et al., 2001; Geary et al., 2012) and it has been considered to be the hallmark of children with dyscalculia (American Psychiatric Association, 2013).

There are two ways to study cognitive determinants in relation to (specific) mathematical skills, i.e., a domain-specific and a domain-general approach (e.g., Fias et al., 2013). Domain-specific approaches investigate the role of number-specific processes, such as the representation of numerical magnitudes in individual differences in mathematics achievement (e.g., De Smedt et al., 2013; Schneider et al., 2016, for a meta-analysis). Domain-general approaches focus on the influence of non-numerical cognitive skills that play a role in mathematical performance, including working memory (e.g., Raghubar et al., 2010; Friso-van den Bos et al., 2013, for a meta-analysis) or executive functions (e.g., Bull and Scerif, 2001; Friso-van den Bos et al., 2013), such as inhibition (e.g., Gilmore et al., 2013).

Over the last 5 years, there has been a large number of studies on individual differences in various mathematical competencies, including arithmetic fact retrieval (e.g., Vanbinst et al., 2012), that has mainly focussed on domain-specific factors, i.e., numerical magnitude processing – or *people’s elementary intuitions about quantity and their ability to understand the meaning of symbolic numbers* (De Smedt et al., 2013; Schneider et al., 2016). In these studies, the role of domain-general factors has been understudied (see for a critique Fias et al., 2013; Szucs et al., 2013; Fias, 2015). Moreover, little attention has been paid to investigating the joint effects of domain-specific factors (e.g., numerical magnitude processing) and domain-general factors. It is also not unlikely that numerical magnitude processing performance itself is also determined, to some extent, by domain-general processes, such as inhibition (Fuhs and McNeil, 2013; Gilmore et al., 2013).

The study of inhibition has recently received some renewed attention in the field of mathematics learning (e.g., Gilmore et al., 2013; Szucs et al., 2013; see also Cragg and Gilmore, 2014, for a review). Inhibition refers to *one’s ability to control one’s attention, behavior, thoughts to override a strong internal predisposition or external lure and instead do what’s more appropriate or needed* (Diamond, 2013, p. 137). Inhibition has been associated with a variety of learning activities (Allan et al., 2014) and inhibitory control early in development appears to be predictive of several outcomes throughout life (Moffitt et al., 2011).

Various studies have shown that inhibition is important for general mathematical development. For example, individual differences in inhibitory control are associated with general mathematical performance in typically developing children (e.g., Bull and Scerif, 2001; Espy et al., 2004; St Clair-Thompson and Gathercole, 2006; Blair and Razza, 2007; Thorell, 2007; Brock et al., 2009; Kroesbergen et al., 2009; Lee et al., 2010; Gilmore et al., 2013; see Allan et al., 2014, for a meta-analysis). It has also been suggested that poor inhibition skills explain part of the low mathematical performance in children with developmental dyscalculia (e.g., Bull and Scerif, 2001; Passolunghi and Siegel, 2004; Szucs et al., 2013), and in children with attentional deficit hyperactivity disorder (ADHD) – which is characterized by poor response inhibition – in which arithmetic deficits have been reported (Kaufmann and Nuerk, 2006). On the other hand, at the neural level the consistent activation increases in the (ventrolateral) prefrontal cortex during mathematical problem solving have been interpreted as reflecting the role of inhibitory control in the solution of these problems (e.g., Cho et al., 2012; Willoughby et al., 2012; Allan et al., 2014; Menon, 2015, for a review). However, several studies failed to find an association between inhibition and math performance (e.g., van der Sluis et al., 2007; Keller and Libertus, 2015). Therefore, the association between inhibition and mathematical performance remains unclear.

One major limitation of the above-reviewed studies that investigated the association between inhibition and mathematical performance is that they investigated mathematics performance with broad general standardized achievement tests. Yet, as suggested by Cragg and Gilmore (2014), the association between inhibition and mathematical skills is likely to vary depending on the mathematical skill under investigation. As our main interest is arithmetic fact retrieval, focussing on inhibition is very relevant, because the association between inhibition and mathematics achievement might be particularly prominent in the context of arithmetic fact retrieval (Verguts and Fias, 2005). Because of the number of features they share, arithmetic facts are particularly prone to interference (De Visscher and Noël, 2013). During arithmetic fact retrieval incorrect but competing answers have to be inhibited, as arithmetic facts are stored in an associative network in semantic memory (e.g., Ashcraft, 1987; Campbell, 1995; Verguts and Fias, 2005). For example, when retrieving the answer to 6 × 3, the incorrect but competing answers to 6 × 2 and 6 × 4, and 5 × 3 and 4 × 3 have to be inhibited. This associative confusion effect is commonly assumed to reflect interference effects (Censabella and Noël, 2007). Therefore, poor inhibition skills can lead to making specific errors when solving these arithmetic fact retrieval problems (e.g., 6 × 3 = 24). Having good inhibition, children are able to inhibit irrelevant associations more efficiently and thus are less likely to develop incorrect associations between problems and their answers (LeFevre et al., 2013). Retrieval difficulties of children with dyscalculia might therefore be related to inefficient inhibition of irrelevant associations (Geary et al., 2012).

Importantly, inhibition is not a unitary concept. Often two main types of inhibition are distinguished: behavioral inhibition or response inhibition (measured with go/no-go tasks or stop-signal tasks) and cognitive inhibition or interference control (measured with Stroop tasks; Diamond, 2013). In this study, we focus on cognitive inhibition. The rationale for focussing on cognitive inhibition is twofold. Firstly, we aim to investigate *cognitive* determinants of individual differences in arithmetic fact retrieval. Secondly, our focus on inhibition is guided by a functional analysis of our task of interest, i.e., arithmetic fact retrieval, or more specifically, by the fact that when children are retrieving arithmetic facts from their memory, competing answers need to be inhibited. To investigate this theoretically appealing link, measures of cognitive inhibition, rather than behavioral inhibition, were selected.

### The Present Study

Against the background of the studies reviewed above, the present study investigated the association between inhibition and arithmetic fact retrieval. We extended the existing body of evidence by focusing on one specific mathematical skill, i.e., arithmetic fact retrieval, and by also studying the joint influence of one other domain-specific skill, i.e., numerical magnitude processing, that has been robustly related to individual differences in mathematics achievement (Schneider et al., 2016), and to arithmetic fact retrieval in particular (Vanbinst et al., 2012, 2015a,b). This allowed us to investigate the unique roles of inhibition and numerical magnitude processing in explaining variability in arithmetic fact retrieval.

Inhibition was measured with cognitive tasks as well as with a questionnaire, because both types of measures capture unique and important aspects of inhibition (e.g., Valiente et al., 2010; Allan et al., 2013). Because we were interested in cognitive determinants of individual differences in arithmetical fact retrieval, we mainly focused on cognitive inhibition – i.e., the ability to supress competing mental representations (Diamond, 2013). We used a numerical and a non-numerical stroop task to measure cognitive inhibition, which allowed us to compare performance on stroop tasks with and without numerical inhibition requirement. We used teacher ratings because teacher ratings of inhibition are more associated with measures of academic skills than parent ratings, given that teachers observe children’s behavior in relation to academic tasks in the classroom (e.g., Blair and Razza, 2007). Combining both measures of inhibition in an experimental setting with the researcher (i.e., by means of stroop tasks) as well as measures of how inhibition skills are reflected in a busy classroom where learning takes place, allowed us to capture the inhibition skills of the children across different settings.

Numerical magnitude processing was measured with symbolic and non-symbolic numerical magnitude comparison tasks, which allowed us to compare performance on numerical tasks with and without symbolic processing requirement (Schneider et al., 2016). We additionally administered a test of intellectual ability and a motor reaction time task to exclude the possibility that associations between our variables of interest and arithmetic fact retrieval were explained by these confounds. We assessed children in the third grade, because we wanted to study the association between arithmetic fact retrieval and inhibition in children who had already acquired a considerable number of arithmetic facts.

To examine the association between arithmetic fact retrieval and inhibition skills, we ran correlational, regression and Bayesian analyses. The use of frequentist analyses allowed us to explore our data by means of a well-known method to gage statistical support for the hypotheses of interest. However, using this *p*-value null hypothesis significance testing has a number of statistical limitations (e.g., Andraszewicz et al., 2015). For example, *p*-values cannot quantify evidence in favor of a null hypothesis, they only signal the extremeness of the data under the null hypothesis and *p*-value logic resembles a proof by contradiction (i.e., low *p*-values indicate extreme data and usually lead researchers to reject the null hypothesis and interpret this as evidence in favor of the alternative hypothesis; Andraszewicz et al., 2015). Unlike null-hypothesis testing, Bayesian statistics allow for the testing of the degree of support for a hypothesis. This is expressed as the Bayes factor, which is the ratio between the evidence in support of the null hypothesis over the alternative hypothesis. By comparing the fit of the data under the null hypothesis to the alternative hypothesis, Bayes factors quantify the evidence in favor of these hypotheses ranging from ‘no evidence’ to ‘extreme evidence’ (see Andraszewicz et al., 2015, for a classification scheme). By adding these analysis we deepen our findings from the traditional regression analysis, as we are able to identify which predictors are the strongest.

Drawing on previous work, we expected that both measures of inhibition skills and measures of numerical magnitude processing would positively correlate with arithmetic fact retrieval skills (the better the inhibition/numerical magnitude processing skills, the better the arithmetic fact retrieval skills). Secondly we verified if both inhibition and numerical magnitude processing play a unique role in arithmetic fact retrieval.

## Materials and Methods

### Participants

Participants were recruited from four elementary schools located in provincial towns in the middle of Flanders, Belgium and had dominantly middle- to high socio-economic background. None of them had a developmental disorder or mental retardation, nor repeated a grade. Initially, 102 children were invited to participate, but the parents of seven children did not give consent. Due to technical problems during data collection, the final sample comprised 86 typically developing third-graders (46 boys, 40 girls) between 8 years 3 months and 9 years 2 months (*M* = 8 years 9 months; *SD* = 4 months). For all participants, written informed parental consent was obtained.

### Materials

Materials consisted of standardized tests, paper-and-pencil tasks and computer tasks designed with E-Prime 2.0 (Schneider et al., 2002). All computer tasks were conducted on a 17-inch notebook computer. Stimuli occurred in white on a black background (Arial font, 72-point size). Response keys were always “d” (left response; labeled with a green sticker) and “k” (right response; labeled with a red sticker). The children were instructed to keep their index fingers on both keys during the task and to perform both accurately and fast. Both accuracy and reaction time (ms) were registered by the computer.

#### Inhibition

Cognitive inhibition was assessed by means of two stroop tasks. We also administered a teacher questionnaire, i.e., the BRIEF (Smidts and Huizinga, 2009), as more ecologically valid measure of inhibition.

##### Stroop tasks

**FIGURE 1:**
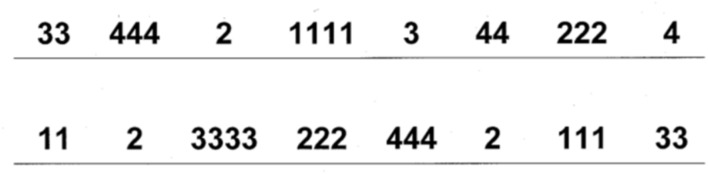
**Stimuli of the numerical stroop task**.

We used two measures of interference control, i.e., Stroop tasks (MacLeod, 1991), in which the processing of possibly interfering information had to be inhibited. We administered a numerical (counting) and a non-numerical (color-word) variant of the Stroop task (van der Sluis et al., 2004). Both tasks involved a baseline and an interference condition and both conditions were preceded by 16 practice trials to ensure that the children understood the task. In the baseline condition the children had to name the number of objects (e.g., how many in ΔΔΔ; ranging from 1 to 4 triangles) in the numerical stroop and name color words (i.e., blue, yellow, red, and green) in the non-numerical stroop. In the interference condition, the children had to name the number of objects (e.g., how many digits in 222; number of objects ranging from 1 to 4; see **Figure 1** for all stimuli) in the numerical stroop and name the ink of a color word (e.g., blue written in red ink) in the non-numerical stroop. Because both the enumeration of stimuli within the subitizing range and the reading of short, well-known words are very automatic processes, the stimuli of both tasks are ideally suitable to measure inhibition by means of a stroop task (which requires an automatic process in the baseline condition for a reliable measure). Each task included four stimuli that were repeated 10 times, namely combinations of the numbers 1, 2, 3, and 4 (see **Figure 1**) in the numerical and combinations of the colors blue, yellow, red, and green in the non-numerical stroop task. All stimuli were presented on a paper, with five lines of eight stimuli. Task administration was the same in both stroop tasks. The child had to name all the stimuli, while the experimenter registered accuracy and time to name the entire sheet. For each condition an inverse efficiency score (i.e., reaction time divided by accuracy) was calculated by dividing the time needed to name the sheet by the accuracy. An index of inhibition was calculated for each task by subtracting the baseline inverse efficiency score from the interference condition inverse efficiency score.

##### BRIEF

The inhibition subscale of the Behavior Rating Inventory of Executive Function or BRIEF (Smidts and Huizinga, 2009) version for teachers was used as a complementary, more ecologically valid measure of children’s inhibition in the classroom. The BRIEF is a standardized questionnaire that consists of 75 items that describe executive functioning behavior, divided in eight subscales (e.g., inhibition, cognitive flexibility, working memory). We used the 10 items of the inhibition subscale (Cronbach α = 0.94), which includes items such as ‘Has difficulties controlling his/her behavior.’ The teacher rated every item for every child on a 3-point scale (never – sometimes – always). The answer ‘never’ scored 1 point, ‘sometimes’ 2 points and ‘always’ 3 points. The score consisted of the sum of the points on the 10 items (max = 30). Higher scores indicated more teacher-reported difficulties in inhibition.

#### Numerical Magnitude Processing

To investigate the role of inhibition in addition to the well-established influence of numerical magnitude processing, we assessed children’s numerical magnitude processing using a symbolic and a non-symbolic numerical magnitude comparison task consisting of Arabic digits and dot arrays, respectively. The tasks consisted of comparing two simultaneously presented numerical magnitudes arranged on either side of the center of the screen. The children had to select the numerically larger magnitude by pressing the key on the side of the larger numerical magnitude. The stimuli in both tasks comprised all combinations of numerosities 1 to 9, yielding 72 trials for each task. Three practice trials were presented for each task. Per task, the stimuli were randomly divided into two blocks and children were given short breaks between blocks. Each trial started with a 200 ms fixation point in the center of the screen and after 1000 ms the stimulus appeared. In the symbolic task, stimuli remained visible until response. In the non-symbolic task, stimuli disappeared after 840 ms in order to avoid counting of the dots. The position of the largest numerosity was counterbalanced. The non-symbolic stimuli were generated with the MATLAB script provided by Piazza et al. (2004) and were controlled for non-numerical parameters (i.e., density, dot size and total occupied area). On half of the trials dot size, array size and density were positively correlated with number, and on the other half they were negatively correlated. These visual parameters were manipulated to ensure that children could not reliably use these non-numerical cues or perceptual features to make a correct decision.

#### Arithmetic Fact Retrieval

Arithmetic fact retrieval was assessed by means of two single-digit verification tasks: one addition task and one multiplication task. Stimuli were selected from a standard set of single-digit arithmetic problems (LeFevre et al., 1996), which excludes tie problems (e.g., 4 + 4) and problems containing 0 and 1 as an operand or answer. The addition items comprised all combinations of the numbers 2 to 9 (*n* = 28) and each item was once presented with the correct answer and once with an incorrect answer, yielding 56 trials. The multiplication items consisted of all items with a product smaller or equal to 25 (*n* = 30), because these small problems are more likely to be solved by direct retrieval from long-term memory (e.g., Campbell and Xue, 2001). Each item was once presented with the correct answer and once with an incorrect answer, yielding 60 trials. The position of the numerically largest operand was counterbalanced. The false solutions in the addition task were created by adding or subtracting 1 or 2 to the solution. The false solutions in the multiplication task were table related, i.e., one of the operands -1 or +1 (*n* = 10; e.g., 6 × 3 = 24), the answer of the corresponding addition (*n* = 10; e.g., 8 × 2 = 10) or unrelated (*n* = 10; e.g., 8 × 3 = 25). Half of the false solutions were numerically larger than the correct answer. Each task was preceded by eight practice trials to familiarize the child with the task requirements. Each trial started with a 250 ms fixation point in the center of the screen and after 750 ms the stimulus appeared. The stimuli remained visible until response. The children had to indicate if the presented solution for the problem was correct (by pressing the left response key, labeled with a green sticker) or false (by pressing the right response key, labeled with a red sticker). For each task, stimuli were presented randomly divided into two blocks and children were given short breaks between blocks.

#### Control Measures

##### Intellectual ability

Raven’s Standard Progressive Matrices (Raven et al., 1992) was used as a measure of intellectual ability (Cronbach α = 0.88). The children were administered 60 multiple-choice items where they had to complete a pattern. The raw score was the number of correct answers within 40 min. For each child a standardized score (*M* = 100, *SD* = 15) was calculated.

##### Motor reaction time

A motor reaction time task was included as a control for children’s response speed on the keyboard. Two shapes, one of which was filled, were simultaneously displayed, one on the left and one on the right of the computer screen. The children had to press the key corresponding to the side of the filled figure. All shapes were similar in size. The administered shapes were circle, triangle, square, star, and heart. Each shape occurred four times filled and four times non-filled. This resulted in 20 trials. Three practice trials were included to familiarize the child with the task. The position of the correct answer (filled shape) was counterbalanced. Each trial started with a 200 ms fixation point in the center of the screen and after 1000 ms the stimulus appeared. The stimuli remained visible until response.

### Procedure

All children were tested at their own school during regular school hours. They all completed three sessions: an individual session (20 min), a session in small groups of four children (45 min), and a group-administered session (60 min). The individual session and small group session with the computer took place in a quiet room. All children were tested in the middle of the third grade. The individual session consisted of both stroop tasks, the small group session of the computer tasks (i.e., motor reaction time, both verification tasks, both comparison tasks), and the group session of the measure of intellectual ability. All children completed the tasks in the same order.

## Results

We explored all data for potential outliers (defined as values larger than three times the interquartile range). In total, nine outliers were identified. Importantly, we analyzed all our data both with and without outliers. Including the outliers did not affect the results. Consequently, all subsequent results are based on the full dataset.

In the stroop and computerized tasks, accuracy was very high [*M* = (0.84–0.99)]. We therefore combined for all these tasks the accuracy and response times into an inverse efficiency score, by dividing a child’s response time by its accuracy. These inverse efficiency scores were included in all subsequent analyses.

There were several careful manipulations built in our tasks. In order to verify if our task format worked, we did several analyses. Firstly, we verified if the task design of the stroop tasks had worked by comparing the baseline and interference condition. As mentioned before we used a difference score as an index of inhibition (calculated by subtracting the baseline score from the interference condition score). This score was significantly different from zero for both the numerical [*t*(85) = 29.24, *p* < 0.001] and the non-numerical [*t*(85) = 22.51, *p* < 0.001] stroop task, which indicates that the task manipulation worked. The results of our analyses were very similar if we calculated (partial) correlations with the interference condition controlled for the baseline condition instead of the difference score.

Secondly, the use of the MATLAB script provided by Piazza et al. (2004) for the non-symbolic numerical magnitude comparison task, including a control for the non-numerical parameters density, dot size and total occupied area, allowed us to divide the items into congruent items (i.e., items where children could reliably use non-numerical cues or perceptual features to make a correct decision, i.e., congruency between visual cues and numerical cues) and incongruent items (i.e., items where children could not reliably use non-numerical cues or perceptual features to make a correct decision, i.e., visual and numerical cues lead to opposite decisions). Our data showed an effect of congruency: congruent items were solved faster than incongruent items [*t*(85) = -4.87; *p* < 0.001]. However, the associations of both types of items with fact retrieval were very similar (for congruent items *r* = 0.27, *p* = 0.012; for incongruent items *r* = 0.22, *p* = 0.044), and therefore, the effect of this manipulation was not further considered.

Thirdly, on the verification tasks we analyzed whether performance on items with different types of false solutions differed in reaction time and/or accuracy. In the addition task accuracy was significantly lower on the items with the false solutions with a distance of 2 of the correct solution, than on the items with a false solution with a distance of 1 of the correct solution (*p* < 0.01). In multiplication, accuracy was significantly lower on the table related false solutions than on the uncorrelated false solutions (*p* < 0.01). We further investigated whether performance on different types of false solutions was differently related to our measures of inhibition. No differential associations of the different types of false solutions with inhibition were found. Consequently, in the following analyses we did not differentiate between the different types of false solutions in the arithmetic verification tasks.

### Descriptive Analyses

The means, standard deviations and ranges for all administered measures are displayed in **Table 1.** The data were well distributed and there were no floor- or ceiling effects.

**Table 1:** Descriptive statistics of the administered measures.

	*N*	*M*	*SD*	Range
**Inhibition**				
Numerical stroop (s)°	86	20.96	6.65	(7.59–38.50)
Non-numerical stroop (s)°	86	25.64	10.56	(8.23–72.08)
BRIEF (raw score)	86	14.08	4.50	(10–27)
**Numerical magnitude processing**				
Symbolic (ms)°	86	908.81	232.72	(602.47–1583.62)
Non-symbolic (ms)°	86	798.64	196.26	(531.82–1439.98)
**Arithmetical fact retrieval**				
Addition verification task (ms)°	86	4099.60	1391.31	(2156.59–8788.95)
Multiplication verification task (ms)°	86	3627.99	2066.55	(1558.79–14420.11)
**Control measures**				
Raven (standardized total score)	86	109.29	10.95	(72–130)
Motor reaction time (ms)°	86	620.81	177.71	(358.89–1158.05)


*°Inverse efficiency score (i.e., response time/accuracy).*

### Correlational Analyses

Pearson correlation coefficients were calculated to examine the associations between the different variables under study (**Table 2**). Correlations with computerized tasks were controlled for motor reaction time by means of partial correlation coefficients. Because of the significant correlation between the arithmetic fact measures (|*r*| = 0.74, *p* < 0.01), we combined these scores (i.e., both verification tasks) into one composite score (i.e., *verification tasks*; consisting of the mean of the verification tasks scores) to improve clarity. This score was included in all subsequent analyses. However, when the pattern of correlations was investigated for each arithmetic fact measure separately, results were very similar.

**Table 2:** Correlation coefficients between the administered measures.

	1	2	3	4	5
(1) Numerical stroop					
(2) Non-numerical stroop	0.25^∗^				
(3) BRIEF	-0.05	0.13			
(4) Symbolic NMP	0.18^a^	0.29^a∗∗^	0.16^a^		
(5) Non-symbolic NMP	0.04^a^	0.24^a∗^	0.16^a^	0.55^a∗∗^	
(6) Verification tasks	0.11^a^	0.18^a^	-0.02^a^	0.61^a∗∗^	0.26^a∗^


*NMP, numerical magnitude processing. ^a^Controlled for motor reaction time. ^∗^*p* < 0.05, ^∗∗^*p* < 0.01.*

All experimental measures that were thought to measure the same underlying component – i.e., the numerical magnitude processing tasks and the inhibition tasks – were significantly correlated with each other, except for the behavioral measure of inhibition (i.e., BRIEF questionnaire), which was not significantly correlated with the two cognitive measures of inhibition (i.e., stroop tasks).

Both numerical magnitude processing tasks were significantly correlated with arithmetic verification, indicating that children with better numerical magnitude processing skills showed better arithmetic fact retrieval performance. This association was stronger for symbolic than for non-symbolic numerical magnitude processing. The William-Steiger test indicated that the difference between these associations was statistically significant [*t*(85) = -4.23, *p* < 0.001].

There was no significant correlation of the behavioural measure of inhibition (BRIEF) with any other measure. We found no significant correlations between the stroop tasks and arithmetic verification. The non-numerical stroop task was significantly correlated with symbolic as well as non-symbolic numerical magnitude processing.

### Regression Analyses

Regression analyses were calculated to assess the unique contribution of numerical magnitude processing and inhibition to arithmetic verification. To this end, all these predictors as well as confounding variables, such as intellectual ability and motor reaction time were entered simultaneously into a regression. In addition, in order to quantify evidence in favor of our hypotheses, Bayes factors were calculated for each predictor with the Bayes Factor package of Morey et al. (2015) implemented in R.

The results of our regression analysis are presented in **Table 3.** Inhibition skills did not significantly predict arithmetic fact retrieval above numerical magnitude processing (*p*s > 0.05). Symbolic numerical magnitude processing significantly predicted fact retrieval (*p* < 0.001), even when controlling for each inhibition task. Non-symbolic numerical magnitude processing did not significantly predict arithmetic fact retrieval (*p* = 0.407).

**Table 3:** Regression analysis of verification and Bayes factors.

Variable	β	*t*	*p*	Bayes factor
Motor reaction time	-0.10	-01.01	0.316	0.41
Intellectual ability	0.08	0.91	0.364	0.31
Numerical stroop	-0.01	-0.05	0.957	0.35
Non-numerical stroop	-0.01	-0.07	0.943	0.76
Symbolic NMP	0.70	6.23^∗∗^	0.000	13 376 272
Non-symbolic NMP	-0.09	-0.83	0.407	2.91


*NMP, numerical magnitude processing. ^∗^*p* < 0.05, ^∗∗^*p* < 0.01.*

Since *p*-values only signal the extremeness of the data under the null hypothesis and *p*-value logic resembles a proof by contradiction – i.e., low *p*-values indicate extreme data and usually lead researchers to reject the null hypothesis and interpret this as evidence in favor of the alternative hypothesis (Andraszewicz et al., 2015) – we also calculated Bayes factors, which are reported in **Table 3.** These factors compare the fit of the data under the null hypothesis compared to the alternative hypothesis, and thereby quantify the evidence in favor of these hypotheses. Our results suggested that the data were more likely to occur under a model including no effect for inhibition (Bayes factor 0.35 for numerical stroop and 0.76 for non-numerical stroop) than a model that included inhibition.

**FIGURE 2:**
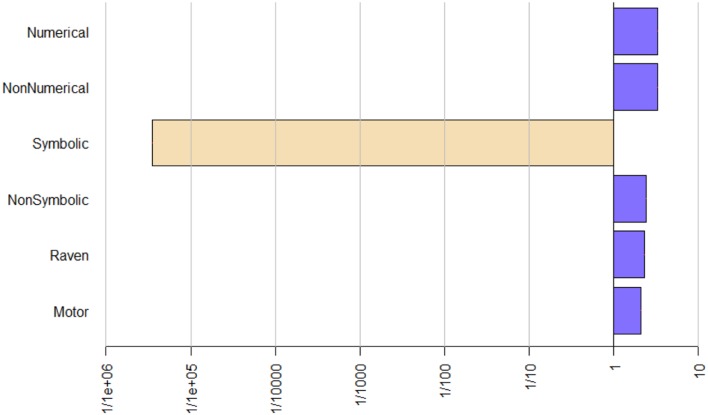
**Change in Bayes factor when a variable is omitted from the model.** The lower the values, the worse the fit of the model to the data. Numerical, numercial stroop; Non-numerical, non-numerical stroop; Symbolic, symbolic numerical magnitude processing; Non-symbolic, non-symbolic numerical magnitude processing; Motor, motor reaction time.

Turning to the effects of numerical magnitude processing, the Bayes factors only provide anecdotal evidence for the hypothesis that non-symbolic numerical magnitude processing is an important predictor. On the other hand, the Bayes factors provide extreme evidence for the hypothesis that symbolic numerical magnitude processing is an important predictor of the variability in arithmetic fact retrieval. As pictured in **Figure 2**, when symbolic numerical magnitude processing is omitted from our regression model, the model loses much of its fit to the data. This is the only predictor where this is the case, which indicates that symbolic numerical magnitude processing is by far the most powerful predictor in our model.

## Discussion

Understanding which cognitive processes underlie individual differences in mathematical skills is a necessary prerequisite to design validated diagnostic tests and appropriate interventions. Numerical magnitude processing has been pointed out as an important factor of these individual differences (Schneider et al., 2016), but the predominant focus on numerical magnitude processing has recently been criticized (Fias et al., 2013; Fias, 2015). On the other hand, several studies provided evidence for an association between inhibition and individual differences in mathematics achievement (Allan et al., 2014, for a meta-analysis). However, it is currently not clear how this association with inhibition occurs in specific aspects of mathematical achievement, such as arithmetic fact retrieval. Importantly, it also needs to be verified whether the association between inhibition and mathematics achievement remains when other crucial factors that have been shown to contribute to individual differences in mathematical competence, such as numerical magnitude processing, are controlled for. Such analysis also reveals whether the well-established association between numerical magnitude processing and mathematical competence is not merely explained by domain-general factors, such as inhibition (Fuhs and McNeil, 2013; Gilmore et al., 2013). We addressed these questions by examining the association between inhibition and arithmetic verification and by investigating the unique contribution of inhibition to arithmetic fact retrieval in addition to numerical magnitude processing.

The present study failed to observe a significant association between inhibition and arithmetic fact retrieval. Moreover, the Bayesian analysis provided evidence for no influence of inhibition. These findings are not in line with the theoretically postulated association between arithmetic fact retrieval and inhibition (e.g., Barrouillet et al., 1997; Geary et al., 2000; Verguts and Fias, 2005; Geary, 2010; Geary et al., 2012) and contradict previous studies that showed an association between inhibition and mathematics achievement (e.g., Bull and Scerif, 2001; Geary et al., 2012; LeFevre et al., 2013; Szucs et al., 2013).

This inconsistency might be explained by various factors. Firstly, it could be due to differences in the measurement of inhibition in the present study, compared to existing studies. For example, LeFevre et al. (2013) used the broad concept *executive attention*, which they defined as the common aspects of executive function and working memory that are necessary in many complex cognitive tasks, including inhibition of competing responses, goal maintenance, and response selection. These authors used span tasks and a color trail test to measure executive attention. These measures tap into broader executive functions rather than inhibition *per se*, which might explain the differences between their findings and the current study. Geary et al. (2012) measured inhibition by counting the number of intrusion errors on an arithmetic task. As a result, these authors derived an index of inhibition from the mathematical task under study, but they did not use an independent measure of inhibition as we did. This measurement difference might again explain differences in results.

Secondly, the inconsistency might be due to the differences in samples that were used. For example, Szucs et al. (2013) showed that children with dyscalculia performed significantly more poorly on several inhibition measures (including the number stroop), pointing to an inhibition deficit in these children. However, the current sample comprised typically developing children. Indeed, it is possible that inhibition has a role in arithmetic fact retrieval in children with mathematical disabilities but not in typically developing children, which could explain the differences between the results of Szucs et al. (2013) and the current study. However, other studies in children with mathematical disabilities did not find significant associations between arithmetic fact retrieval and inhibition. Both van der Sluis et al. (2004) and Censabella and Noël (2007) found that children with mathematical disabilities did not differ from the typically developing children on (the same) inhibition tasks as in our study. The present findings, although only applicable to typically developing children and not to children with mathematical disabilities, are in line with these studies that failed to find a significant association between inhibition and mathematical performance. Moreover, recently Keller and Libertus (2015) also failed to find an association between inhibition on general mathematical skills in typically developing 5- and 6-year-olds. The current data extend the results of existing studies by specifically focussing on the theoretically appealing association between inhibition and arithmetic fact retrieval, i.e., because during arithmetic fact retrieval incorrect but competing answers have to be inhibited, as arithmetic facts are stored in an associative network in semantic memory. Importantly, it is unclear whether the children with mathematical disabilities in van der Sluis et al. (2004) and Censabella and Noël (2007) had specific problems with arithmetic fact retrieval. For example, Censabella and Noël (2007) stated that, although assumed, their sample of children with mathematical disabilities did not have difficulties in arithmetic fact retrieval *per se*. Therefore, these studies should be replicated in samples of children with specific difficulties in arithmetic fact retrieval.

The present study also investigated the association between numerical magnitude processing and arithmetic fact retrieval. We used both symbolic and non-symbolic tasks to verify whether the access to numerical magnitudes from symbolic digits or numerical magnitude processing *per se* is related to arithmetic fact retrieval (see De Smedt et al., 2013, for a discussion). In line with Vanbinst et al. (2012, 2015a,b) and De Smedt et al. (2013), the importance of symbolic numerical magnitude processing in arithmetic fact retrieval was supported by our results. More specifically, we found a unique association between symbolic numerical magnitude processing and fact retrieval. Thus, children with better symbolic numerical magnitude processing skills showed better arithmetic fact retrieval performance. We also found a significant correlation of non-symbolic numerical magnitude processing with fact retrieval. However, as strongly indicated by our Bayesian analysis, symbolic numerical magnitude is a much stronger predictor than non-symbolic numerical magnitude.

Recently, Fuhs and McNeil (2013) and Gilmore et al. (2013) suggested that performance on the non-symbolic numerical magnitude processing task is determined by domain-general processes such as inhibition. We also explored this issue in our data. However, the results on the association between inhibition and the non-symbolic numerical magnitude processing task were not so univocal. We observed an association with the non-numerical stroop task, but found no association with the numerical stroop task. On the other hand, we also found an association between the non-numerical stroop task and the symbolic numerical magnitude processing task. However, these associations did not explain the association between numerical magnitude processing and arithmetical fact retrieval.

### Limitations and Future Directions

Firstly, our choice for specific measures of inhibition might have had an important impact on our findings. The present study only included one specific measure of inhibition, i.e., Stroop tasks, yet inhibition is not a unitary construct, but a family of functions (Harnishfeger, 1995; Dempster and Corkill, 1999; Hasher et al., 1999; Nigg, 2000; Shilling et al., 2002; Censabella and Noël, 2007). Different aspects of inhibitory control are dissociable from each other at both the behavioral and the neural levels (Diamond, 2013). One often-used distinction between different types of inhibition is between behavioral inhibition and cognitive inhibition (Diamond, 2013). Different tasks (e.g., Stroop tasks, Flanker task, go/no-go task, stop-signal task) are used to measure these different types of inhibition, and their association with arithmetic fact retrieval might be different. Future studies should therefore use a combination of different measurements of inhibition. Additionally, there are several versions of the Stroop task [e.g., animal stroop (Szucs et al., 2013), spatial stroop (Diamond, 2013), numerical stroop (Bull and Scerif, 2001)], and, in particular, of the numerical stroop task. We used a counting stroop task as numerical stroop task. The difference between the different versions of the tasks lies in the information that has to be inhibited. In our numerical stroop task (i.e., Counting Stroop Task), the number represented by the digits had to be inhibited in favor of the quantity of digits in the array. On the other hand, in the Number Stroop (e.g., Kaufmann and Nuerk, 2006) magnitudes of two one-digit numbers who differ in physical size are compared and participants have to inhibit the irrelevant physical size in favor of the numerical magnitude of the digits (or vice versa). Because these tasks contain different kinds of numerical information that needs to be inhibited (i.e., the number represented by digits vs. the physical size of the digit), they may be differently related to arithmetic fact retrieval. Moreover, the design of the classic stroop task we used in this study might have had an impact on the performance on the task, as children see all stimuli at once, which can influence cognitive load, i.e., higher load when seeing the stimuli all at once vs. a trial-by-trial administration (e.g., Kindt et al., 1996). Future studies could compare the design we used (i.e., a card format) to a design where one stimulus at a time is presented (e.g., a computer task), in order to investigate the impact of the design on performance on the task.

Importantly, we also included a teacher questionnaire, the BRIEF (Smidts and Huizinga, 2009) to measure inhibition skills in children, but this measure was not associated with our measures of cognitive inhibition. Although the inhibition subscale of the BRIEF is known to be reliable, the use of 10 items might have been limited to capture sufficient intersubject variability. Moreover, it could be due to measure selection, namely the stroop task being a direct measure of inhibition and the BRIEF being an indirect measure. It could also be due to the task design of the stroop task (e.g., number of trials). On the other hand, the current data are in line with several studies who failed to observe a significant correlation between ratings (e.g., BRIEF questionnaire) and performance-based measures (e.g., stroop tasks), suggesting these measures assess different aspects of inhibition (Mahone et al., 2002; McAuley et al., 2010; Toplak et al., 2013).

Secondly, the participant selection in this study might have had an impact on our results. We only investigated children in third grade, which might explain why we did not find an association between inhibition skills and arithmetic fact retrieval. Although children in third grade have already acquired a considerable number of arithmetic facts, there is still room for improvement in automatizing these facts. Through the course of primary school, problem-answer associations become stronger, and more efficient arithmetic fact retrieval arises (Vanbinst et al., 2015a). Moreover, inhibitory control also continues to mature through the course of primary school (Luna et al., 2004; Luna, 2009). On the other hand, inhibition could play a role in the learning of arithmetic facts, because similarity between arithmetic facts provokes interference and this could lead to difficulties in storing arithmetic facts in long-term memory (De Visscher and Noël, 2014a,b). Therefore, it would be interesting for future studies to investigate the association between inhibition and fact retrieval in children of different ages. Additionally, the present study comprised typically developing children. It might be that the association between inhibition and arithmetic fact retrieval is only observed in the context of atypical development of arithmetic fact retrieval and/or of atypical development of inhibition. Future studies should examine the association between inhibition and arithmetic fact retrieval skills in atypical groups, such as children with arithmetic fact retrieval deficits and children with ADHD – who are known to have deficits in (response) inhibition (e.g., Bayliss and Roodenrys, 2000; Kaufmann and Nuerk, 2006).

Thirdly, the association between inhibition and mathematics observed in previous studies might also be explained by other factors that are associated with both individual differences in mathematics and inhibition. Potential examples include working memory (Friso-van den Bos et al., 2013; Peng et al., 2015), socio-economic status and home environment (Sarsour et al., 2011; Dilwordth-Bart, 2012; Keller and Libertus, 2015). These factors should be considered in future studies.

Future studies should also examine the association between inhibition and arithmetic fact retrieval at the neural level. Even though this association might not be detectable at the behavior level, it might be that it can be observed at the neural level. Indeed, neuroimaging data might generate findings that cannot be detected by behavioral data alone (De Smedt et al., 2010). Brain areas associated with inhibition (e.g., prefrontal cortex) are often found to be activated during mathematical tasks (e.g., Menon, 2015, for a review). Although many fMRI studies have pointed to prefrontal cortex control processes during arithmetic fact retrieval (Menon, 2015), there is no study that has directly investigated the overlap between these control networks and arithmetic fact retrieval. Cho et al. (2012) found that increased retrieval use was correlated with the dorsolateral and ventrolateral prefrontal cortex, areas that are also known to show increased activity during inhibition. The authors suggested that this increase in the lateral prefrontal cortex suggested the involvement of inhibitory processes, yet they did not directly test this hypothesis. Future studies should investigate this hypothesis with imaging studies, for example by investigating the neural overlap between an arithmetic task and an inhibition localizer task.

## Author Contributions

EB and BS developed the study concept. EB developed the test materials, collected the data and performed the data analysis. EB, WF, and BS drafted the manuscript. WF and BS provided critical feedback to different versions of the manuscript.

## Conflict of Interest Statement

The authors declare that the research was conducted in the absence of any commercial or financial relationships that could be construed as a potential conflict of interest.

## References

[B1] Allan N. P. Hume L. E. Allan D. M. Farrington A. L. Lonigan C. J. (2014). Relations between inhibitory control and the development of academic skills in preschool and kindergarten: a meta-analysis. *Dev. Psychol.* 50 2368–2379. 10.1037/a0037493 25069051

[B2] Allan N. P. Lonigan C. J. Wilson S. B. (2013). Psychometric evaluation of the children’s behavior questionnaire – very short form in preschool children using parent and teacher report. *Early Child. Res. Q.* 28 302–313. 10.1016/j.ecresq.2012.07.009

[B3] American Psychiatric Association (2013). *Diagnostic and Statistical Manual of Mental Disorders*, 5th Edn Washington, DC: American Psychiatric Association.

[B4] Ancker J. S. Kaufman D. (2007). Rethinking health numeracy: a multidisciplinary literature review. *J. Am. Med. Inform. Assoc.* 14 713–721. 10.1197/jamia.M2464 17712082 PMC2213486

[B5] Andraszewicz S. Scheibehenne B. Rieskamp J. Grasman R. Verhagen J. Wagenmakers E. J. (2015). An introduction to Bayesian hypothesis testing for mangagement research. *J. Manag. Res.* 41 521–543.

[B6] Ashcraft M. H. (1987). “Children’s knowledge of simple arithmetic: a developmental model and simulation,” in *Formal Methods in Developmental Research*, eds Brainerd C. J. Kail R. Bisanz J. (New York, NY: Springer-Verlag), 302–338.

[B7] Barrouillet P. Fayol M. Lathulière E. (1997). Selecting between competitors in multiplication tasks: an explanation of the errors produced by adolescents with learning disabilities. *Int. J. Behav. Dev.* 21 253–275. 10.1080/016502597384857

[B8] Bayliss D. M. Roodenrys S. (2000). Executive processing and ADHD: an application of the supervisory attentional system. *Dev. Neuropsychol.* 17 161–180. 10.1207/S15326942DN1702_02 10955201

[B9] Blair C. Razza R. P. (2007). Relating effortful control, executive function, and false belief understanding to emerging math and literacy ability in kindergarten. *Child Dev.* 78 647–663. 10.1111/j.1467-8624.2007.01019.x 17381795

[B10] Brock L. L. Rimm-Kaufman S. E. Nathanson L. Grimm K. J. (2009). The contribution of ‘hot’ and ‘cool’ executive function to children’s academic achievement, learning-related behaviors, and engagement in kindergarten. *Early Child. Res. Q.* 24 337–349. 10.1016/j.ecresq.2009.06.001

[B11] Bull R. Scerif G. (2001). Executive functioning as a predictor of children’s mathematics ability: inhibition, switching, and working memory. *Dev. Neuropsychol.* 19 273–293. 10.1207/S15326942DN1903_3 11758669

[B12] Campbell J. I. D. (1995). Mechanisms of simple addition and multiplication: a modified network-interference theory and simulation. *Math. Cogn.* 1 121–164.

[B13] Campbell J. I. D. Xue Q. L. (2001). Cognitive arithmetic across cultures. *J. Exp. Psychol. Gen.* 130 299–315. 10.1037/0096-3445.130.2.299 11409105

[B14] Censabella S. Noël M. P. (2007). The inhibition capacities of children with mathematical disabilities. *Child Neuropsychol.* 14 1–20. 10.1080/09297040601052318 18097799

[B15] Cho S. Metcalfe A. W. S. Young C. B. Ryali S. Geary D. C. Menon V. (2012). Hippocampal-prefrontal engagement and dynamic causal interactions in the maturation of children’s fact retrieval. *J. Cogn. Neurosci.* 24 1849–1866. 10.1162/jocn_a_00246 22621262 PMC3462165

[B16] Cragg L. Gilmore C. (2014). Skills underlying mathematics: the role of executive function in the development of mathematics proficiency. *Trends Neurosci. Educ.* 3 63–68. 10.1016/j.tine.2013.12.001

[B17] De Smedt B. Ansari D. Grabner R. H. Hannula M. M. Schneider M. Verschaffel L. (2010). Cognitive neuroscience meets mathematics education. *Educ. Res. Rev.* 5 97–105. 10.1016/j.edurev.2009.11.001

[B18] De Smedt B. Noël M. P. Gilmore C. Ansari D. (2013). The relationship between symbolic and non-symbolic numerical magnitude processing skills and the typical and atypical development of mathematics: a review of evidence from brain and behavior. *Trends Neurosci. Educ.* 2 48–55. 10.1016/j.tine.2013.06.001

[B19] De Visscher A. Noël M. P. (2013). A case study of arithmetic facts dyscalculia caused by a hypersensitivity-to-interference in memory. *Cortex* 49 50–70. 10.1016/j.cortex.2012.01.003 22321390

[B20] De Visscher A. Noël M. P. (2014a). Arithmetic facts storage deficit: the hypersensitivity-to-interference in memory hypothesis. *Develop. Sci.* 17 434–442. 10.1111/desc.12135 24410798

[B21] De Visscher A. Noël M. P. (2014b). The detrimental effect of interference in multiplication facts storing: typical development and individual differences. *J. Exp. Psychol. Gen.* 143 2380–2400. 10.1037/xge0000029 25347536

[B22] Dempster F. N. Corkill A. J. (1999). Individual differences in susceptibility to interference and general cognitive ability. *Acta Psychol.* 101 395–416. 10.1016/S0001-6918(99)00013-X

[B23] Diamond A. (2013). Executive Functions. *Annu. Rev. Psychol.* 64 135–168. 10.1146/annurev-psych-113011-143750 23020641 PMC4084861

[B24] Dilwordth-Bart J. E. (2012). Does executive function mediate SES and home quality associations with academic readiness? *Early Child. Res. Q.* 27 416–425. 10.1016/j.ecresq.2012.02.002

[B25] Dowker A. (2005). *Individual Differences in Arithmetic. Implications for Psychology, Neuroscience and Education.* Hove: Psychology Press.

[B26] Espy K. A. McDiarmid M. M. Cwik M. F. Stalets M. M. Hamby A. Senn T. E. (2004). The contribution of executive functions to emergent mathematic skills in preschool children. *Dev. Neuropsychol.* 26 465–486. 10.1207/s15326942dn2601_6 15276905

[B27] Fias W. (2015). “Neurocognitive components of mathematical skills and dyscalculia,” in *Development of Mathematical Cognition: Neural Substrates and Genetic Influences*, eds Berch D. Geary D. Mann Koepke K. (Amsterdam: Elsevier), 195–217.

[B28] Fias W. Menon V. Szucs D. (2013). Multiple components of development dyscalculia. *Trends Neurosci. Educ.* 2 43–47. 10.1016/j.tine.2013.06.006

[B29] Finnie R. Meng R. (2001). Cognitive skills and the youth labour market. *Appl. Econ. Lett.* 8 675–679. 10.1080/13504850110037877

[B30] Friso-van den Bos I. van der Ven S. H. G. Kroesbergen E. H. van Luit J. E. H. (2013). Working memory and mathematics in primary school children: a meta-analysis. *Educ. Res. Rev.* 10 29–44. 10.1016/j.edurev.2013.05.003

[B31] Fuhs M. McNeil N. (2013). ANS acuity and mathematics ability in preschoolers from low-income homes: contributions of inhibitory control. *Dev. Sci.* 16 136–148. 10.1111/desc.12013 23278935

[B32] Geary D. C. (2010). Mathematical disabilities: reflections on cognitive, neuropsychological, and genetic components. *Learn. Individ. Diff.* 20 130–133. 10.1016/j.lindif.2009.10.008 PMC2821095 20161681

[B33] Geary D. C. Hamson C. O. Hoard M. K. (2000). Numerical and arithmetical cognition: a longitudinal study of process and concept deficits in children with learning disability. *J. Exp. Child Psychol.* 77 236–263. 10.1006/jecp.2000.2561 11023658

[B34] Geary D. C. Hoard M. K. Bailey D. H. (2012). Fact retrieval deficits in low achieving children and children with mathematical learning disability. *J. Learn. Disabil.* 45 291–307. 10.1177/0022219410392046 21252374 PMC3163113

[B35] Gilmore C. Attridge N. Clayton S. Cragg L. Johnson S. Marlow N. (2013). Individual differences in inhibitory control, not non-verbal number acuity, correlate with mathematics achievement. *PLoS ONE* 8:e67374 10.1371/journal.pone.0067374 PMC3681957 23785521

[B36] Harnishfeger K. K. (1995). “The development of cognitive inhibition: theories, definitions, and research evidence,” in *Interference and Inhibition in Cognition*, eds Dempster F. N. Brainerd C. J. (San Diego, CA: Academic Press), 175–204.

[B37] Hasher L. Zacks R. T. May C. P. (1999). “Inhibitory control, circadian arousal, and age,” in *Attention and Performance XVII. Cognitive Regulation of Performance: Interaction of Theory and Application*, eds Gopher D. Koriat A. (Cambridge, MA: The MIT Press), 653–675.

[B38] Kaufmann L. Nuerk H. C. (2006). Interference effects in a numerical stroop paradigm in 9- to 12-year-old children with ADHD-C. *Child Neuropsychol.* 12 223–243. 10.1080/09297040500477483 16837397

[B39] Keller L. Libertus M. (2015). Inhibitory control may not explain the link between approximation and math abilities in kindergarteners from middle class families. *Front. Psychol.* 6:685 10.3389/fpsyg.2015.00685 PMC4440905 26052306

[B40] Kilpatrick J. Swafford J. Findell B. (2001). *Adding It Up: Helping Children Learn Mathematics.* Washington, DC: National Academies Press.

[B41] Kindt M. Bierman D. Brosschot J. F. (1996). Stroop versus stroop: comparison of a card format and a single-trial format of the standard color-word stroop task and the emotional stroop task. *Pers. Individ. Dif.* 21 653–661. 10.1016/0191-8869(96)00133-X

[B42] Kroesbergen E. Van Luit J. Van Lieshout E. Van Loosbroek E. Van der Rijt B. (2009). Individual differences in early numeracy: the role of executive functions and subitizing. *J. Psychoeduc. Assess.* 27 226–236. 10.1177/0734282908330586

[B43] Lee K. Ng S. F. Pe M. L. Ang S. Y. Hasshim M. N. A. M. Bull R. (2010). The cognitive underpinnings of emerging mathematical skills: executive functioning, patterns, numeracy, and arithmetic. *Br. J. Educ. Psychol.* 82 82–99. 10.1111/j.2044-8279.2010.02016.x 22429059

[B44] LeFevre J. A. Berrigan L. Vendetti C. Kamawar D. Bisanz J. Skwarchuk S. L. (2013). The role of executive attention in the acquisition of mathematical skills for children in Grades 2 through 4. *J. Exp. Child Psychol.* 114 243–261. 10.1016/j.jecp.2012.10.005 23168083

[B45] LeFevre J. A. Sadesky G. S. Bisanz J. (1996). Selection of procedures in mental addition: reassessing the problem size effect in adults. *J. Exp. Psychol. Learn. Mem. Cogn.* 22 216–230.

[B46] Luna B. (2009). Developmental changes in cognitive control through adolescence. *Adv. Child Dev. Behav.* 37 233–278. 10.1016/S0065-2407(09)03706-9 19673164 PMC2782527

[B47] Luna B. Garver K. E. Urban T. A. Lazar N. A. Sweeney J. A. (2004). Maturation of cognitive processes from late childhood to adulthood. *Child Dev.* 75 1357–1372. 10.1111/j.1467-8624.2004.00745.x 15369519

[B48] MacLeod C. (1991). Half a century of research on the Stroop effect: an integrative review. *Psychol. Bull.* 109 163–203. 10.1037/0033-2909.109.2.163 2034749

[B49] Mahone E. M. Cirino P. T. Cutting L. E. Cerrone P. M. Hagelthorn K. M. Hiemenz J. R. (2002). Validity of the behaviour rating inventory of executive function in children with ADHD and/or Tourette syndrome. *Arch. Clin. Neuropsychol.* 17 643–662. 10.1016/S0887-6177(01)00168-8 14591848

[B50] McAuley T. Chen S. Goos L. Schachar R. Crosbie J. (2010). Is behavior rating inventory of executive function more strongly associated with measures of impairment or executive function? *J. Int. Neuropsychol. Soc.* 16 495–505. 10.1017/S1355617710000093 20188014

[B51] Menon V. (2015). “Arithmetic in the Child and Adult Brain,” in *The Oxford Handbook of Mathematical Cognition*, eds Cohen-Kadosh R. Dowker A. (Oxford: Oxford University Press), 502–530.

[B52] Moffitt T. E. Arseneault L. Belsky D. Dickson N. Hancox R. J. Harrington H. (2011). A gradient of childhood self-control predicts health, wealth, and public safety. *Proc. Natl. Acad. Sci. U.S.A.* 108 2693–2698. 10.1073/pnas.1010076108 21262822 PMC3041102

[B53] Morey R. D. Rouder J. N. Jamil T. (2015). *BayesFactor: Computation of Bayes Factors for Common Designs. R Package Version 0.9.9.*

[B54] Nigg J. T. (2000). On inhibition/disinhibition in developmental psychopathology: views from cognitive and personality psychology and a working inhibition taxonomy. *Psychol. Bull.* 126 220–246. 10.1037/0033-2909.126.2.220 10748641

[B55] Passolunghi M. C. Siegel L. S. (2004). Working memory and access to numerical information in children with disability in mathematics. *J. Exp. Child Psychol.* 88 348–367. 10.1016/j.jecp.2004.04.002 15265681

[B56] Peng P. Namkung J. Barnes M. Sun C. (2015). A meta-analysis of mathematics and working memory: moderating effects of working memory domain, type of mathematics skill, and sample characteristics. *J. Educ. Psychol.* 108 455–473. 10.1037/edu0000079

[B57] Piazza M. Izard V. Pinel P. Le Bihan D. Dehaene S. (2004). Tuning curves for approximate numerosity in the human intraparietal sulcus. *Neuron* 44 547–555. 10.16/j.neuron.2004.10.014 15504333

[B58] Raghubar K. Barnes F. Hecht S. (2010). Working memory and mathematics: a review of developmental, individual difference, and cognitive approaches. *Learn. Individ. Diff.* 20 110–122. 10.1016/j.lindif.2009.10.005

[B59] Raven J. C. Court J. H. Raven J. (1992). *Standard Progressive Matrices.* Oxford: Oxford Psychologists Press.

[B60] Ritchie S. J. Bates T. C. (2013). Enduring links form childhood mathematics and reading achievement to adult socioeconomic status. *Psychol. Sci.* 24 1301–1308. 10.1177/0956797612466268 23640065

[B61] Sarsour K. Sheridan M. Jutte D. Nuru-Jeter A. Hinshaw S. Boyce W. T. (2011). Family socioeconomic status and child executive functions: the roles of language, home environment, and single parenthood. *J. Int. Neuropsychol. Soc.* 17 120–132. 10.1017/S1355617710001335 21073770

[B62] Schneider M. Beeres K. Coban L. Merz S. Schmidt S. S. Stricker J. (2016). Associations of non-symbolic and symbolic numerical magnitude processing with mathematical competence: a meta-analysis. *Dev. Sci.* 10.1111/desc.12372 [Epub ahead of print]. 26768176

[B63] Schneider W. Eschmann A. Zuccolotto A. (2002). *E-Prime Reference Guide.* Pittsburgh, PA: Psychology Software Tools.

[B64] Shilling V. M. Chetwynd A. Rabbitt P. M. A. (2002). Individual inconsistency across measures of inhibition: an investigation of the construct validity of inhibition in older adults. *Neuropsychologia* 40 605–619. 10.1016/S0028-3932(01)00157-9 11792402

[B65] Smidts D. P. Huizinga M. (2009). *BRIEF Executieve Functies Gedragsvragenlijst: Handleiding [BRIEF Executive Functions Behavior Inventory].* Amsterdam: Hogrefe Uitgevers.

[B66] St Clair-Thompson H. Gathercole S. (2006). Executive functions and achievement in school: shifting, updating, inhibition, and working memory. *Q. J. Exp. Psychol.* 59 745–759. 10.1080/17470210500162854 16707360

[B67] Szucs D. Devine A. Soltesz F. Nobes A. Gabriel F. (2013). Developmental dyscalculia is related to visuo-spatial memory and inhibition impairment. *Cortex* 49 2674–2688. 10.1016/j.cortex.2013/06.007 23890692 PMC3878850

[B68] Thorell L. B. (2007). Do delay aversion and executive function deficits make distinct contributions to the functional impact of ADHD symptoms? A study of early academic skill deficits. *J. Child Psychol. Psychiatry* 48 1061–1070. 10.1111/j.1469-7610.2007.01777.x 17995481

[B69] Toplak M. E. West R. F. Stanovich K. E. (2013). Practioner review: do performance-based measures and ratings of executive function assess the same construct? *J. Child Psychol. Psychiatry* 54 131–143. 10.1111/jcpp.12001 23057693

[B70] Valiente C. Lemery-Chalfant K. Swanson J. (2010). Prediction of kindergartners’ academic achievement from their effortful control and emotionality: evidence for direct and moderate relations. *J. Educ. Psychol.* 102 550–560. 10.1037/a0018992

[B71] van der Sluis S. de Jong P. F. van der Leij A. (2004). Inhibition and shifting in children with learning deficits in arithmetic and reading. *J. Exp. Child Psychol.* 87 239–266. 10.1016/j.jecp.2003.12.002 14972600

[B72] van der Sluis S. de Jong P. F. van der Leij A. (2007). Executive functioning in children, and its relations with reasoning, reading, and arithmetic. *Intelligence* 35 427–449. 10.1016/j.intell.2006.09.001

[B73] Vanbinst K. Ceulemans E. Ghesquière P. De Smedt B. (2015a). Profiles of children’s arithmetic fact development: a model-based clustering approach. *J. Exp. Child Psychol.* 133 29–46. 10.1016/j.jecp.2015.01.003 25731679

[B74] Vanbinst K. Ghesquière P. De Smedt B. (2015b). Does numerical processing uniquely predict first graders’ future development of single-digit arithmetic? *Learn. Individ. Diff.* 37 153–160. 10.1016/j.lindif.2014.12.004

[B75] Vanbinst K. Ghesquière P. De Smedt B. (2012). Numerical magnitude representations and individual differences in children’s arithmetic strategy use. *Mind Brain Educ.* 6 129–136. 10.1111/J.1751-228X.2012.01148.x

[B76] Verguts T. Fias W. (2005). Interacting neighbors: a connectionist model of retrieval in single-digit multiplication. *Mem. Cogn.* 33 1–16. 10.3758/BF03195293 15915789

[B77] Willoughby M. T. Kupersmidt J. B. Voegler-Lee M. E. (2012). Is preschool executive function causally related to academic achievement? *Child Neuropsychol.* 18 79–91. 10.1080/09297049.2011.578572 21707258 PMC3417807

